# Poster Session I - A164 THE ASSOCIATION OF INFLAMMATORY BOWEL DISEASE WITH CARCINOID TUMORS: A CASE-BASED SYSTEMATIC REVIEW

**DOI:** 10.1093/jcag/gwaf042.164

**Published:** 2026-02-13

**Authors:** N He, K Dong, B Nguyen

**Affiliations:** Medicine, The University of British Columbia, Vancouver, BC, Canada; Medicine, The University of British Columbia, Vancouver, BC, Canada; Medicine, The University of British Columbia, Vancouver, BC, Canada

## Abstract

**Background:**

Carcinoid tumors are a relatively rare subset of neuroendocrine tumors that commonly arise in the gastrointestinal (GI) tract. Inflammatory Bowel Disease (IBD), including Crohn’s disease and Ulcerative Colitis (UC), is associated with an increased risk of certain malignancies, but its relationship with carcinoid tumors is poorly defined. Evidence to date is limited to case reports, and there has yet to be a review that has comprehensively summarized this association.

**Aims:**

To investigate the association of IBD with carcinoid tumors through a systematic review of published case reports and series.

**Methods:**

We performed a systematic review according to PRISMA guidelines, searching MEDLINE. Descriptive statistics were performed on patient demographics and treatment outcomes.

**Results:**

A total of 38 articles (33 case reports, 5 case series) from 1981 to 2025 were selected for analysis. A total of 46 individual cases of patients with IBD and co-existing carcinoid tumors were extracted. Crohn’s disease accounted for 30/46 (65.22%) and UC accounted for 16/46 (34.78%) of total cases. The mean duration of IBD prior to discovery of carcinoid was 15.05 years, and the mean age at IBD diagnosis was 45.2 years. 5-ASA use was the most common treatment option (43.48%), followed by combination therapy of 5-ASA and steroids (21.74%). Other treatment options included steroids only, biologics, and immunosuppressants. The mean treatment duration prior to carcinoid discovery was 9.21 years. The majority of carcinoid tumors were found in the appendix (30.43%), ileum (28.26%), and rectum (19.57%). Most tumors were well-differentiated and had a mean diameter of 21.48 mm. The method of discovery of the carcinoid was similar with 27/46 (58.70%) cases being found symptomatically and 19/46 (41.30%) cases being found incidentally.

**Conclusions:**

This review of 46 reported cases of carcinoid tumors occurring in patients with IBD highlights a potential association between chronic inflammation and the development of GI carcinoid tumors. A substantial minority were detected incidentally, highlighting the need for clinical vigilance. Larger multicenter studies are needed to capture the link between IBD and neuroendocrine tumorigenesis and identify high-risk patients.

A160 Table 1: Summary of Carcinoid Tumor Characteristics and IBD Context

**Funding Agencies:**

None

**Table:** 

Category	Variable	Frequency
Carcinoid Tumor Location	Appendix	14/46 (30.43%)
	Ileum	13/46 (28.26%)
	Rectum	9/46 (19.57%)
	Colon	5/46 (10.87%)
	Lung	3/46 (6.52%)
	Duodenum	2/46 (4.35%)
Tumor Characteristics	Well-differentiated	22/36 (88.89%)
	Poorly differentiated	4/36 (11.11%)
IBD Treatment History	5-ASA alone	10/23 (43.48%)
	5-ASA + steroids	5/23 (21.74%)
	Steroids only	3/23 (13.04%)
	Biologics	3/23 (13.04%)
	Immunosuppressants	2/23 (8.70%)

